# Powdered tart cherry supplementation mitigates the post-exercise immune response with reduction in total antioxidant status and serum triglyceride levels following an acute bout of intense endurance exercise

**DOI:** 10.1186/1550-2783-11-S1-P34

**Published:** 2014-12-01

**Authors:** C Goodenough, K Levers, R Dalton, E Galvan, A O’Connor, S Simbo, N Barringer, J Carter, C Seesselberg, A Coletta, YP Jung, M Koozehchian, B Sanchez, S Springer, M Cho, S Mertens-Talcott, C Rasmussen, M Greenwood, R Kreider

**Affiliations:** 1 Texas A&M University, College Station, Texas, USA

## Background

Consumption of tart cherry juice has been reported to effectively reduce inflammation, muscle damage, and muscle soreness following bouts of exercise. The purpose of this study was to determine if consumption of a powdered form of tart cherries derived from tart cherry skins prior to and following intense endurance exercise promotes similar positive results as seen with tart cherry juice consumption.

## Methods

27 endurance trained or triathlete (21.8±3.9 yr, 15.0±6.0% body fat, 67.4±11.8 kg) men (n=18) and women (n=9) volunteered to participate in this study and were matched based on average reported race pace, age, body weight, and fat free mass. Subjects were randomly assigned to ingest, in a double blind manner, capsules containing a placebo (P, n=16) or powdered tart cherries (CherryPURE® Freeze Dried Tart Cherry Powder [TC, n=11]). Participants ingested the supplements one time daily (480 mg/d) for 10-d including day of exercise up to 48-hr post-exercise. A half-marathon run (13.1 mi/21.1 km) was completed under 2-hr (111.98±11.9 min) as the intense endurance exercise protocol. Fasting blood samples were taken pre-run, 60-minutes post-run as well as after 24 and 48 hours of recovery and analyzed by MANOVA with repeated measures. Consent to publish the results was obtained from all participants.

## Results

Overall changes in markers of general immune response, WBC and LYMPH, were observed in both groups over time (p<0.001), but the overall Wilks’ Lambda MANOVA analysis did not reveal a significant group x time effect (p=0.22) for these markers. MANOVA univariate analysis revealed significant effects for LYMPH and WBC (p<0.001) in both groups over time. A significant group x time quadratic effect was shown for WBC (p=0.034) in addition to a trend toward a significant delta value based on group assignment for WBC (p=0.09). The overall Wilks’ Lambda MANOVA analysis did not reveal a significant time (p=0.78) or group x time effect (p=0.32) for SOD and TAS. MANOVA univariate analysis revealed a significant group x time linear effect for TAS (p=0.046) in addition to a trend toward a significant delta value based on group assignment for TAS (p=0.099). No significant group x time interaction was evident with SOD (p=0.73). Overall changes in RBC, HCT, TG, and TotCHL were observed in both groups over time (p<0.001), but the overall MANOVA analysis did not reveal a significant group x time effect (p=0.438). MANOVA univariate analysis revealed significant group x time cubic effect for TG (p=0.043). No significant group x time interaction was evident with RBC (p=0.46), HCT (p=0.48), and TotCHL (p=0.73).

## Conclusion

Results of this study indicate that short-term supplementation with powdered tart cherries over the 7 days leading up to, during, and 2 days after intense endurance exercise aid in reduction of the general immune response as indicated by a significantly lower WBC response post-exercise compared to a placebo supplement. The mitigated immune response following exercise with powdered tart cherry supplementation correlates with the decreased catabolic response indicated by BUN/Cr ratio and cortisol levels reported in a companion abstract. Further, as a result of powdered tart cherry supplementation compared to a placebo, the TAS response was also significantly lower suggesting a diminished release of ROS and RNS in response to the endurance exercise bout. With powdered tart cherry supplementation compared to a placebo, blood triglyceride levels were also significantly reduced over the post-exercise recovery period. Overall, these findings suggest that supplementation with a powdered tart cherry product surrounding an intense endurance event reduces the general immune and free radical response typically correlated with endurance exercise in addition to reducing serum triglyceride levels. Further research is necessary to determine long-term supplementation effects with endurance training.

